# Salivary metabolite profiling distinguishes patients with oral cavity squamous cell carcinoma from normal controls

**DOI:** 10.1371/journal.pone.0204249

**Published:** 2018-09-20

**Authors:** Pawadee Lohavanichbutr, Yuzheng Zhang, Pei Wang, Haiwei Gu, G. A. Nagana Gowda, Danijel Djukovic, Matthew F. Buas, Daniel Raftery, Chu Chen

**Affiliations:** 1 Program in Epidemiology, Division of Public Health Sciences, Fred Hutchinson Cancer Research Center, Seattle, Washington, United States of America; 2 Program in Biostatistics and Biomathematics, Division of Public Health Sciences, Fred Hutchinson Cancer Research Center, Seattle, Washington, United States of America; 3 Department of Genetics and Genomics Sciences, Icahn School of Medicine at Mount Sinai, New York, New York, United States of America; 4 Center for Metabolic and Vascular Biology, School of Nutrition and Health Promotion, College of Health Solutions, Arizona State University, Phoenix, Arizona, United States of America; 5 Northwest Metabolomics Research Center, Anesthesiology and Pain Medicine, University of Washington, Seattle, Washington, United States of America; 6 Department of Cancer Prevention and Control, Roswell Park Comprehensive Cancer Center, Buffalo, New York, United States of America; 7 Translational Research Program, Division of Public Health Sciences, Fred Hutchinson Cancer Research Center, Seattle, Washington, United States of America; 8 Department of Epidemiology, School of Public Health, University of Washington, Seattle, Washington, United States of America; 9 Department of Otolaryngology-Head and Neck Surgery, School of Medicine, University of Washington, Seattle, Washington, United States of America; Mayo Clinic Rochester, UNITED STATES

## Abstract

Oral cavity squamous cell carcinoma (OCC) and oropharyngeal squamous cell carcinoma (OPC) are among the most common cancers worldwide and are associated with high mortality and morbidity. The purpose of this study is to identify potential biomarkers to distinguish OCC/OPC from normal controls and to distinguish OCC patients with and without nodal metastasis. We tested saliva samples from 101 OCC, 58 OPC, and 35 normal controls using four analytical platforms (NMR, targeted aqueous by LC-MS/MS, global aqueous and global lipidomics by LC-Q-TOF). Samples from OCC and normal controls were divided into discovery and validation sets. Using linear regression adjusting for age, sex, race and experimental batches, we found the levels of two metabolites (glycine and proline) to be significantly different between OCC and controls (FDR < 0.1 for both discovery and validation sets) but did not find any appreciable differences in metabolite levels between OPC and controls or between OCC with and without nodal metastasis. Four metabolites, including glycine, proline, citrulline, and ornithine were associated with early stage OCC in both discovery and validation sets. Further study is warranted to confirm these results in the development of salivary metabolites as diagnostic markers.

## Introduction

Squamous cell carcinoma of the oral cavity and oropharynx (OSCC) is associated with high case-fatality. In addition, OSCC and its treatment often lead to life-long impairment of orofacial function and to pain and disfigurement. World-wide, an estimate of 529,000 new cases and 292,000 deaths occurred in 2012 (http://globocan.iarc.fr/Default.aspx) [1, 2]. In the US, 49,670 new cases and 9,700 deaths are estimated to occur in 2017 [3]. OSCC accounts for about 75% of the head and neck squamous cell cancers (HNC); about two thirds of the OSCC are oral cavity cancers and one third are oropharyngeal cancers. For oral cavity cancers (OCC), with tobacco smoking and alcohol abuse as the principal etiologic factors, the overall 5-year survival is about 70% for stage I or II disease, 45% for stage III disease and about 35% for stage IV disease (http://www.cancer.org). For oropharyngeal cancer (OPC), infection with human papillomavirus (HPV, mostly HPV-16) is most likely the etiologic factor in about two thirds of these patients. The 5-year survival for patients with HPV-positive OPC is about 80–85%, which is considerably better than that for OCC patients and HPV-negative OPC patients [4–8]. These data point to the importance to detect OCC and OPC early. However, there is a lack of early detection biomarkers for both OCC and OPC.

At present, the classification of OSCC to inform treatment or prognosis is heavily dependent on AJCC stage. Yet, the ability of staging to inform treatment and predict prognosis is limited; patients with tumors of the same clinical and pathologic staging have a heterogeneous response to clinical treatment, and different probability of recurrence and survival [9]. The treatment can vary from unimodality treatment (surgery or radiation) for early stage disease to multimodality treatments (some combination of surgery, radiation, and chemotherapy) for late stage disease. While metastasis to the cervical lymph nodes is the single most important independent predictor of survival [10, 11], the current clinical diagnosis of nodal metastasis relies on physical examination and auxiliary imaging modalities, such as computed tomography (CT), magnetic resonance imaging (MRI), positron emission tomography (PET) and ultrasound, which have limited sensitivities to detect nodal metastasis. Based on the results in a meta-analysis, the sensitivity estimates for CT, MRI, PET, and ultrasound were 52%, 65%, 66%, and 66%, respectively [12]. The current standard of care for patients with T1 tumors (≤2 cm) without clinically apparent neck metastasis based on physical examination and imaging is close follow-up by clinical and radiologic exams. For patients with T2-T4 tumors (>2 cm at the longest dimension), a majority of patients undergo prophylactic neck dissection. In our prior study [13] of OSCC patients treated at the University of Washington Medical Centers, 22% of patients with clinically normal necks without a neck dissection developed nodal metastasis within 18 months post diagnosis; 38% of those patients who underwent neck dissection did not have lymph node metastasis, which is consistent with a prior report [14]. Thus, under the current clinical practice guidelines, a substantial proportion of oral cancer patients are either under-treated or over-treated, pointing to the need to develop a new clinical test to accurately stratify patients according to their nodal metastasis status to inform the necessity for a neck surgery to remove the metastatic lymph nodes.

The field of metabolomics offers a promising alternative approach for the identification of biomarkers associated with the presence of OSCC at its earliest stage or with occult nodal metastasis. Metabolomics describes the study of concentrations and fluxes of low molecular weight (MW) metabolites present in biofluids or tissue that provide detailed information on biological systems and their current status [15–28]. The quantitative analysis of over 1000 small molecules (MW<1000 Da) is now available on a relatively routine basis. Numerous studies have established that the cellular energy metabolism of a broad spectrum of cancers, including oral cancer, is fundamentally altered and that alteration is evident in the metabolite profiles of not only the tumors, but also the patient’s body fluids such as saliva, serum/plasma and urine [22, 24, 29–34]. In particular, the metabolomics profiles in saliva [30, 34], serum [31] or plasma [32] were able to distinguish persons with OSCC from those with precursor lesions such as oral lichen planus and leukoplakia, and from controls without oral pathology. Furthermore, a study of the salivary metabolite profiles for oral, breast and pancreatic cancers found that the metabolomics profiles are cancer type-specific [33]. However, to the best of our knowledge, no prior study has evaluated salivary metabolomics in relation to nodal metastasis in OSCC. Metabolite profiling could potentially represent a novel alternative or adjunct to physical examination and radiologic imaging for accurate detection of nodal metastasis in OSCC patients.

As an attempt to meet the challenges of detecting OSCC early and to better characterize OSCC patients based on their nodal status, the current study used pre-treatment prospectively-collected saliva samples, well-characterized demographic, life style and clinical information, and state-of-the-art metabolomics technologies to examine the metabolite profiles of OSCC patients. It is of particular interest to us to use saliva for this investigation, since saliva is in close contact with cancers in the mouth and can be easily obtained without any invasive procedure.

## Materials and methods

### Ethics statement

This study was conducted with written informed consent of the study participants and the approval of the Institutional Review Boards of the Fred Hutchinson Cancer Research Center and the Veteran Affairs Puget Sound Health Care System.

### Study population

Eligible cases were patients with primary OSCC treated at University of Washington Medical Center, Harborview Medical Center, and the Veterans Affairs Puget Sound Healthcare System in Seattle, Washington from December 2003 to February 2012. Eligible controls were patients without OSCC who had oral surgery such as tonsillectomy at the same institutions where the OSCC patients were treated during the same period. Study participants’ demographic and life style information was obtained through in-person interviews using a structured questionnaire; clinical characteristics were obtained through medical record abstraction, as previously described [35].

### Sample collection and study design

Saliva samples were collected preoperatively. It was requested that patients did not eat or drink anything, except water, within an hour before saliva collection. Saliva was collected using a 50 mL sterile conical centrifuge tube and transferred on ice to the laboratory within two hours of collection. At the laboratory, saliva was spun at 1300 x g in a refrigerated centrifuge at 4° C for 10 minutes to collect all the saliva at the bottom of the tube. The saliva sample was then gently mixed by pipetting up and down and aliquoted into 0.5 ml cryovials before storage in a -80° C freezer.

Saliva samples were tested in two sets of 100 samples each, approximately one year apart in time. One sample in the first set was too viscous to be processed and was excluded. To maximize the opportunity to examine the potential differences in metabolite profiles between patients with and without nodal metastasis, the saliva samples for the first set were selected from OCC patients with known pathological nodal status (45 N+ and 34 N-). The first set also included 20 randomly selected saliva samples from controls. The second set of samples contained saliva samples from 58 OPC patients, 22 OCC patients, and 20 controls (including five control samples that were tested in the first set in order to determine whether there were batch-to-batch variations in the test results).

### Reagents

Acetonitrile (ACN), ammonium acetate, and acetic acid (LC-MS grade) were purchased from Fisher Scientific (Pittsburgh, PA). Sodium phosphate, monobasic (NaH_2_PO_4_), sodium phosphate, dibasic (Na_2_HPO_4_), and 3-(trimethylsilyl)propionic acid-2,2,3,3-d_4_ sodium salt (TSP) were obtained from Sigma-Aldrich (St. Louis, MO). The standard compounds corresponding to the measured metabolites were purchased from Sigma-Aldrich and Fisher Scientific. D_2_O, stable isotope-labeled tyrosine and lactate internal standards (L-tyrosine-^13^C_2_ and sodium-L-lactate-^13^C_3_) were purchased from Cambridge Isotope Laboratories, Inc. (Tewksbury, MA). The purities of non-labeled standards were >95–99%, whereas the purities of the two ^13^C-labeled compounds were > 99%.

### Metabolite profiling

Metabolite profiling analyses using nuclear magnetic resonance (NMR) and three types of liquid chromatography mass spectrometry (LC-MS) were performed at the Northwest Metabolomics Research Center (NWMRC), University of Washington, and are described below.

#### NMR experiments and analysis

For sample preparation, phosphate buffer solution (100 mM) was prepared by dissolving 1,124 mg anhydrous Na_2_HPO_4_ and 249.9 mg anhydrous NaH_2_PO_4_ in 100 g D_2_O. A solution of TSP was added to achieve a final concentration of 50 μM. The pH of the buffer solution was 7.45. Saliva samples were thawed at room temperature and 100 μL of each saliva sample was mixed with 110 μL phosphate buffer (100 mM; pH = 7.45) in a 1.5 ml Eppendorf tube (Fisher Scientific). The mixture was then centrifuged at 19,925 × g for 10 min to remove particulate matter, if any, using an Eppendorf centrifuge and the supernatant was transferred to a 3 mm NMR tube.

^1^H NMR experiments were performed at 298 K on a Bruker Avance III 800 MHz spectrometer equipped with a cryogenically cooled probe and Z-gradients. The CPMG (Carr-Purcell-Meiboom-Gill) pulse sequence with water suppression using presaturation was used for the1D NMR experiments. All NMR spectra were obtained using 32,768 time domain data points, 9615 Hz spectral width and 3 s recycle delay. The data were Fourier transformed with a spectrum size of 131,072 points after multiplying the FID with an exponential window function with a line broadening (LB) of 0.5 Hz. The spectra were then phase and baseline corrected, and chemical shifts were referenced to the internal TSP signal. Bruker Topspin versions 3.1 and 3.2 software packages were used for NMR data acquisition and processing, respectively. Peak assignments were made based on established literature [36, 37], including the human metabolome database (HMDB) [38], and the biological magnetic resonance data bank (BMRB) [39]. A typical NMR spectrum of a saliva sample with peak assignments is shown in S1 Fig. The Bruker AMIX software package version 3 was used to quantify metabolites. Integrals of characteristic, isolated peaks for metabolites in saliva were obtained using AMIX software (Bruker). Relative metabolite concentrations were obtained after normalizing the spectra using the total spectral sum.

#### Targeted LC-MS for aqueous metabolite profiling

Frozen saliva samples were first thawed at 4°C, and 50 μL of each sample was placed into a 2 mL Eppendorf tube. Protein precipitation and metabolite extraction were performed by adding 150 μL of methanol; the mixture was then vortexed for 2 min and stored at -20°C for 20 min. Each sample was then centrifuged at 20,817 x g for 10 min, and 100 μL of the supernatant was collected into a new Eppendorf vial. To the first vial containing the pellet, another 300 μL methanol was added, and the mixture was vortexed for 10 min to allow thorough metabolite extraction. After centrifuging this mixture at 20,817 x g for 10 min, 300 μL of the supernatant was collected into the same vial that contained the previous supernatant. The resulting supernatants from two rounds of extractions were dried using a Vacufuge Plus evaporator (Eppendorf, Hauppauge, NY). The dried samples were reconstituted in 500 μL 5 mM ammonium acetate in 40% water/60% acetonitrile + 0.2% acetic acid containing 5.13 μM L-tyrosine-^13^C_2_ and 22.5 μM sodium-L-lactate-^13^C_3_. The two isotope-labeled internal standards were added to each sample to monitor the system performance. A pooled sample, which was a mixture of all the study samples, was used as the quality control (QC) sample and was analyzed once for every ten study samples.

The robust targeted LC-MS/MS method we developed has been used in a number of studies at the Northwest Metabolomics Research Center (NW-MRC) [40–44]. Briefly, all LC-MS/MS experiments were performed on an Agilent 1260 LC (Agilent Technologies, Santa Clara, CA)-AB Sciex QTrap 5500 mass spectrometer (AB Sciex, Toronto, ON, Canada) system. Each sample was injected twice, 10 μL for analysis using negative ionization mode and 2 μL for analysis using positive ionization mode. Both chromatographic separations were performed in hydrophilic interaction chromatography (HILIC) mode on two Waters XBridge BEH Amide columns (150 x 2.1 mm, 2.5 μm particle size, Waters Corporation, Milford, MA) connected in parallel. The flow rate was 0.300 mL/min, auto-sampler temperature was kept at 4°C, and the column compartment was set at 40°C. The mobile phase was composed of Solvents A (5 mM ammonium acetate in 90%H_2_O/ 10% acetonitrile + 0.2% acetic acid) and B (5 mM ammonium acetate in 90%acetonitrile/ 10% H_2_O + 0.2% acetic acid). After the initial 2 min isocratic elution of 90% B, the percentage of Solvent B decreased to 50% at t = 5 min. The composition of Solvent B maintained at 50% for 4 min (t = 9 min), and then the percentage of B gradually went back to 90%, to prepare for the next injection.

The mass spectrometer is equipped with an electrospray ionization (ESI) source. Targeted data acquisition was performed in multiple-reaction-monitoring (MRM) mode. We monitored 121 and 80 MRM transitions in negative and positive mode, respectively (201 transitions in total). The LC-MS system was controlled by Analyst 1.5 software (AB Sciex). The extracted MRM peaks were integrated using MultiQuant 2.1 software (AB Sciex).

#### Global aqueous and lipidomics LC-MS experiments

The saliva samples were thawed at 4 ^o^C. After vortexing for 20 s, 100 μL of each saliva sample was mixed with 200 μL chloroform:methanol (2:1; v:v). The mixture was vortexed for 2 min and then incubated at -20 ^o^C for 30 min. The mixture was then centrifuged at 20,817 x g for 10 min. One hundred μL of the top layer of each sample was collected into a new 2 mL Eppendorf tube. After drying, it was reconstituted into 100 μL H_2_O/ACN (4:6, v:v) prior to global aqueous metabolomics experiments. In contrast, 100 μL from the bottom layer of each sample was collected and injected for lipidomics experiments.

The aqueous global metabolomics experiments were performed using the Agilent 1200 SL LC-6520 Quadrupole-Time of Flight (Q-TOF) MS system (Agilent Technologies). The separation conditions for the LC-Q-TOF experiments were the same as those for the LC-MS/MS described above. The ESI voltage was 3.8 kV, and the m/z scan range was 60–1000. The Q-TOF data were extracted using Agilent MassHunter Qualitative Analysis (version B.07.00), Quantitative Analysis (version B.07.01), and Mass Profiler Professional (MPP, version B.13.00) software. The absolute intensity threshold for the LC-Q-TOF data extraction was 1000, and the mass accuracy limit was set to 10 ppm.

#### Lipidomics LC-Q-TOF experiments

The lipidomics data were collected using a standard metabolic profiling method and the same Agilent 6520 QTOF-MS platform [45]. Briefly each prepared sample (4 μL for positive ESI ionization, 8 μL for negative ESI ionization) was injected onto an Agilent Zorbax 300 SB-C8 column (2.1× 50mm, 1.8-μm), which was heated to 50°C. The flow rate was 0.4 mL/min. Mobile phase A was 5 mM ammonium acetate and 0.1% formic acid in water, and mobile phase B was 5% water in ACN containing 5 mM ammonium acetate and 0.1% formic acid. The mobile phase composition was kept isocratic at 35% B for 1 min, and was increased to 95% B in 19 min; after another 10 min at 95% B, the mobile phase composition was returned to 35% B. The ESI voltage was 3.8 kV. The Q-TOF MS spectrometer was calibrated prior to each batch, and a reference channel infusing the standard reference mixture (G1969-85001, Agilent Technologies) was used during the experiments to ensure mass accuracy. The mass scan range was 100–1600, and the acquisition rate was 1.5 spectra/s. The Q-TOF data were extracted using Agilent MassHunter Qualitative Analysis (version B.07.00) and Mass Profiler Professional (MPP, version B.13.00) software. The absolute intensity threshold for the LC–Q-TOF data extraction was 1000, and the mass accuracy limit was set to 10 ppm.

### Data processing

We processed and normalized data from each platform (NMR, targeted aqueous, global lipidomics and global aqueous) for each sample set (first and second) separately. Raw data for the first and the second set were provided in S1 Table and S2 Table, respectively. The normalization includes the sample-wise median center assuming the overall abundance across samples are the same. Values were log2 transformed and metabolites that were missing in > 30% of samples were filtered out. We applied K-Nearest Neighbor (KNN) method to impute the missing values. The tuning parameter K was trained by the cross validation with minimum error rate. After data normalization and processing, there were 2,610 metabolite signals (after removing isotope and adduct peaks) retained in the first set and 5,722 metabolite signals retained in the second set. There were 453 metabolites overlapped between the two sets, including 114 from targeted aqueous, 45 from NMR, 66 from global lipidomics, and 228 from the global aqueous platform. Further analyses were limited to these 453 metabolites detected in both sample sets. The normalized data of the 453 metabolites are provided in S3 Table.

### Statistical and metabolite pathway analyses

**Fig 1:**
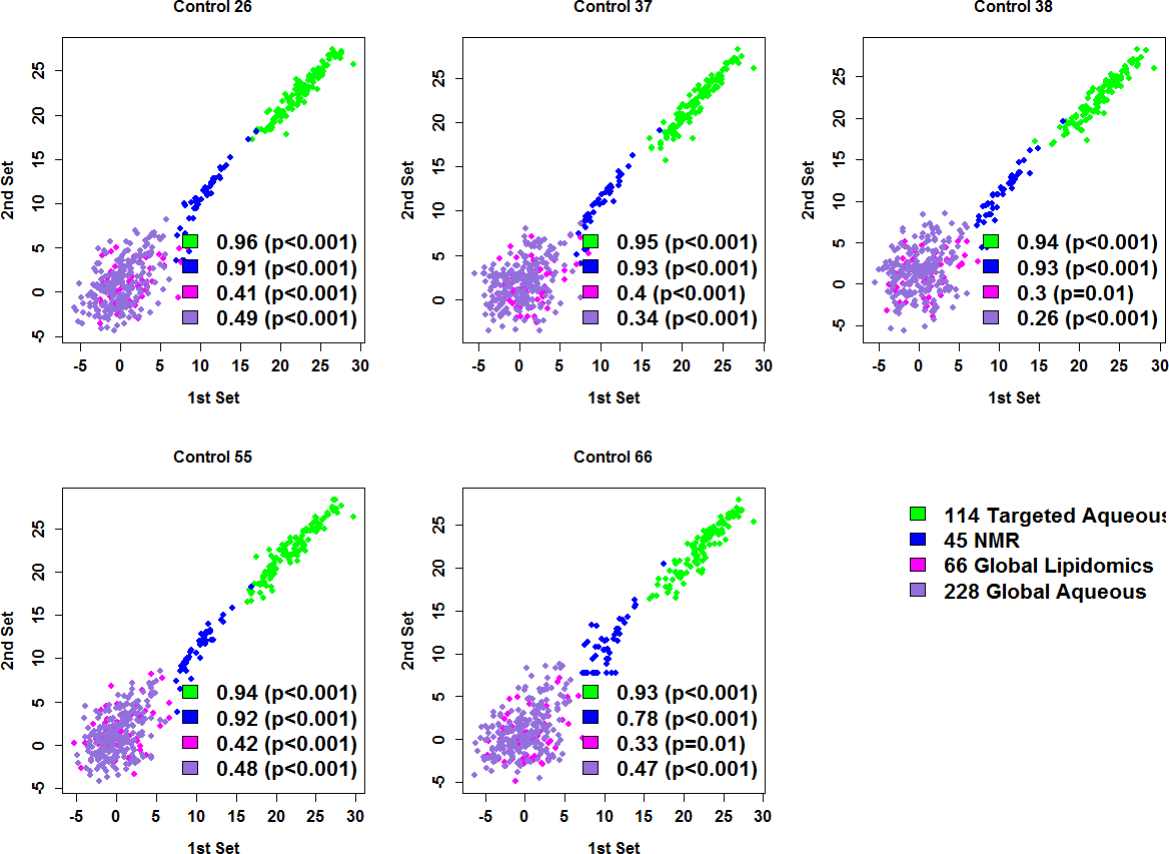
Correlation of the 453 metabolites for the five control samples that were tested in both first and second sets. The graphs showed Pairwise Pearson correlation coefficients and p-values for each type of metabolite profiling for each control.

We first investigated whether we could combine data from the first and the second set by looking at the correlation among metabolites of the five control samples that were tested in both sets. We found good correlation for targeted aqueous profiling (pairwise correlation coefficient 0.93–0.96) and for NMR (pairwise correlation coefficient 0.78–0.93), but poor correlation for global lipidomics (pairwise correlation coefficient 0.3–0.42) and for global aqueous profiling (pairwise correlation coefficient 0.26–0.49) (Fig 1). Ideally, we would randomly split samples into discovery and validation set. However, because of the poor correlations between the two sample sets for global metabolomics tests, it would not be appropriate to combine the two sets and randomly split them. Therefore, we used data of OCC and control samples from the first set for discovery and those from the second set for validation. Since the sample size for OPC is relatively small, we did not divide it into discovery and validation set. Linear regression adjusting for age, sex, race and experimental batch were performed to test the difference between OCC vs. control; early stage T1/T2 (OCC) vs. control; late stage T3/T4 (OCC) vs. early stage T1/T2 (OCC); node-positive OCC vs. node- negative OCC; and OPC vs. control. We further derived False Discovery Rate (FDR) using the Benjamini and Hochberg (BH) method [46] for each platform separately to adjust for multiple comparisons. Pathway analysis, including pathway enrichment and topology analysis, was performed using MetaboAnalyst 3.0 [47].

## Results

Selected clinical characteristics of cases and controls in the study are presented in Table 1. Cases were more likely to be older, current smokers and current drinkers.

**Table 1:** Selected characteristics of study participants.

	First Set		Second Set	
	Case (n = 79)	Control (n = 20)	Case (n = 80)	Control (n = 20)
**Age**				
<50	14 (17.7%)	13 (65.0%)	16 (20.0%)	13 (65.0%)
50–59	24 (30.4%)	4 (20.0%)	37 (46.3%)	3 (15.0%)
60–69	22 (27.8%)	3 (15.0%)	17 (21.3%)	4 (20.0%)
70+	19 (24.1%)	0	10 (12.5%)	0
**Sex**				
F	23 (29.1%)	8 (40.0%)	17 (21.3%)	5 (25.0%)
M	56 (70.9%)	12 (60.0%)	63 (78.8%)	15 (75.0%)
**Race**				
Non-white	8 (10.1%)	3 (15.0%)	8 (10.0%)	4 (20.0%)
White	71 (89.9%)	17 (85.0%)	72 (90.0%)	16 (80.0%)
**History of tobacco smoking**				
Current	37 (46.8%)	5 (35.7%)	28 (35.4%)	5 (29.4%)
Never/Former	42 (53.2%)	9 (64.3%)	51 (64.6%)	12 (70.6%)
Unknown	0	6	1	3
**History of alcohol use**				
Current	52 (66.7%)	8 (57.1%)	53 (68.8%)	11 (64.7%)
Never/Former	26 (33.3%)	6 (42.9%)	24 (31.2%)	6 (35.3%)
Unknown	1	6	3	3
**Tumor Site**				
Oral Cavity	79 (100.0%)	-	22 (27.5%)	-
Oropharynx	0	-	58 (72.5%)	-
**Tumor stage**				
T1/T2	40 (50.6%)	-	51 (68.0%)	-
T3/T4	39 (49.4%)	-	24 (32.0%)	-
Unknown	0	-	5	-
**Nodal Status**				
Negative	34 (43.0%)	-	10 (21.3%)	-
Positive	45 (57.0%)	-	37 (78.7%)	-
Unknown	0	-	33	-

### Workflow and summary of the number of metabolites

**Fig 2:**
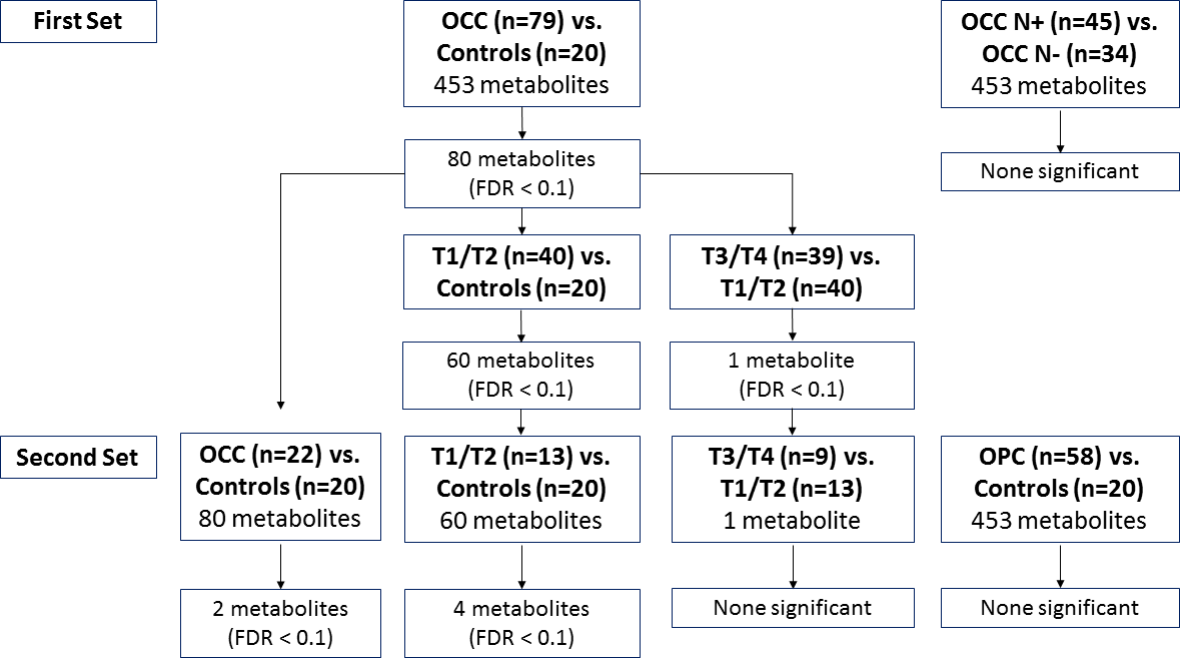
Workflow for the data analyses. The first set was used as discovery and the second set was used as validation for comparing between OCC vs. controls.

Workflow and the number of significant salivary metabolites for each comparison are presented in Fig 2.

We first compared salivary metabolites of OCC patients to those of controls on each platform separately using the data from the first set. The p-values and FDRs for all 453 metabolites were shown in S4 Table. We found the levels of 80 metabolites (30 targeted aqueous, 20 NMR, and 30 global aqueous metabolites) to be different between OCC and controls using FDR < 0.1 as criteria (Table 2). We did not find any metabolites tested on global lipidomics to be significantly different in their levels between OCC and controls. Among the 80 metabolites, the levels of 12 metabolites (glutamine, glycine, glucose, proline, succinate, isoleucine, glutamic acid, lactate, tyrosine, valine, leucine, and alanine) were significantly different as measured by the targeted aqueous and NMR platforms. Three metabolites (proline, glutamine, and lactate) were consistently different between OCC and controls across all three platforms.

**Table 2:** Metabolites differentiating between OCC and controls in the first sample set.

Targeted Aqueous Profiling			NMR			Global Aqueous Profiling		
Compound Name	Coeff*	p-value	Compound Name	Coeff*	p-value	Compound Name	Coeff*	p-value
**α-Ketoglutaric acid**	-1.34	4.89E-06	**Glutamate**	-1.10	3.79E-07	**L-isoleucyl-L-proline**	0.89	2.16E-04
**Glutamine**	-1.39	1.30E-05	**Dimethylamine**	-1.21	1.11E-06	**Proline**	-1.33	4.11E-04
**Glycine**	-1.66	1.19E-05	**Proline**	-1.01	2.19E-05	**Cystathionine ketimine**	0.94	0.001
**Glucose**	1.13	3.40E-05	**2-hydroxy-3-methylvalerate**	0.85	3.74E-04	**Threonate**	1.11	0.001
**Sarcosine**	-2.05	7.30E-05	**Succinate**	1.64	4.19E-04	**C8H20N7O**	-1.24	0.001
**Citrulline**	-1.42	1.24E-04	**Isoleucine**	-0.78	0.001	**Lactate**	1.45	0.001
**Serine**	-0.96	4.46E-04	**Leucine**	-0.83	0.002	**C3H6O3**	1.25	0.001
**Proline**	-1.22	0.001	Trimethylamine	1.35	0.003	**C11H23N3O3**	0.72	0.001
**Succinate**	1.47	0.001	**Glucose**	1.10	0.003	**6-Lactoyltetrahydropterin**	0.71	0.001
**isoLeucine**	-0.89	0.001	**Tyrosine**	-0.60	0.005	**D-Glutamine**	-1.04	0.002
Oxalacetate	-0.86	0.002	2-Hydroxybutyrate	0.86	0.009	**Midodrine**^**#**^	0.63	0.002
Glutamic acid	-0.89	0.003	**Lactate**	1.11	0.010	**N2,N2-Dimethylguanosine**	0.73	0.003
**Agmatine**	-0.71	0.003	**Valine**	-0.62	0.010	**L-Norleucine**	-0.76	0.005
**Ornithine**	-1.02	0.004	**Phenylalanine**	-0.58	0.013	**N-Methyl-D-aspartic acid**	-0.55	0.005
**Glycerate**	-0.62	0.005	Glutamine	-0.69	0.016	**Zanamivir**^**#**^	-0.70	0.006
**Lactate**	0.94	0.005	Glycine	-0.94	0.016	**C16H33N6O8**	0.54	0.006
**12-HETE**	-0.91	0.005	**Mannose**	0.80	0.015	C6H14O3	0.90	0.006
Tyrosine	-0.83	0.006	**Alanine**	-0.63	0.024	**C7H19N4O3**	0.52	0.008
Aspartic Acid	-0.68	0.010	Threonine	-0.68	0.024	**Falaconitine**	0.74	0.008
Lysine	-0.85	0.010	**2-hydroxyisovaleric acid**	0.72	0.027	2,6-Dimethoxyphenol	0.80	0.008
**Oxypurinol**	-0.74	0.010				**Dexpanthenol**^**#**^	0.47	0.009
**Valine**	-0.70	0.013				**Methylripariochromene A**^**#**^	0.86	0.010
Xanthosine	-0.66	0.013				**N-Acetyl-L-Histidine**	-1.11	0.011
**Leucine**	-0.62	0.014				**Hexaflumuron**^**##**^	-0.72	0.011
Pipecolate	-0.81	0.015				**Cellotetraose**	0.95	0.014
Alanine	-0.60	0.016				d-Dethiobiotin	0.86	0.016
**5-Aminovaleric acid**	-0.67	0.019				**C19H35N3O14**	-0.75	0.017
Histidine	-0.80	0.017				**LeucomycinA3**^**#**^	0.47	0.018
Pyruvate	0.61	0.019				**C12H24N2O3**	0.48	0.018
Creatine	-1.01	0.021				Tetradecylsulfate	0.59	0.019

*coefficients of the linear regression adjusting for age, sex, and race

^#^drugs or drug metabolites

^##^insecticide

Metabolites in bold were also different between T1/T2 OCC vs. controls

We further explored which of the 80 metabolites were associated with early stage or late stage tumor by comparing T1/T2 vs. controls and T3/T4 vs. T1/T2. Using FDR < 0.1 as criteria, we found 60 metabolites differentiated between early stage tumor and controls (bolded in Table 2). One of the 80 metabolites (cystathionine ketimine) significantly differentiated early stage from late stage tumor (p-value 0.003, FDR 0.079). We then validate the results in the second set of samples. The case-control differences for two of the 80 metabolites (glycine and proline) were validated in the second sets (Table 3). The association with case-control status remained significant after further adjusting for smoking and drinking status (p-values for glycine and proline were 0.013 and 0.025, respectively). Among the 60 metabolites with differential levels between T1/T2 OCC vs. controls, four metabolites (citrulline, glycine, ornithine, and proline) were also significant in the validation set (Table 3). Further adjusting for smoking and drinking did not significantly change the association between metabolite levels and T1/T2 vs. control status (the respective p-values for glycine, proline, ornithine, and citrulline were 0.002, 0.002, 0.006, and 0.037). Cystathionine ketimine was not different between early and late stage tumors in the validation set (p-value 0.64).

**Table 3:** List of salivary metabolites showing significantly different relative concentrations between oral cavity cancer cases and controls in both first and second sets.

Compound Name	Coefficient (1st set)	p-value (1st set)	FDR (1st set)	Coefficient (2nd set)	p-value (2nd set)	FDR (2nd set)
**Cases vs. Controls**						
Proline	-1.22	7.44E-04	0.011	-1.03	0.003	0.080
Glycine	-1.66	1.19E-05	6.00E-04	-0.95	0.005	0.080
**T1/T2 vs. Controls**						
Proline	-0.78	0.054	0.086	-1.37	0.001	0.016
Glycine	-1.11	0.008	0.030	-1.16	0.003	0.027
Citrulline	-1.34	0.001	0.010	-0.87	0.010	0.064
Ornithine	-0.81	0.047	0.083	-0.89	0.017	0.080

To identify potential markers for nodal metastasis, we compared salivary metabolites among 79 OCC cases with known pathological nodal status (45 N+ vs. 34 N-) using a linear regression analysis adjusting for age, sex, and race. All 79 samples were tested in the same set (first set) since the number was too small to be divided into discovery and validation sets. Twenty-one metabolites were different between N+ and N- (p < 0.05) but none reached statistical significance after adjustment for multiple comparisons (S4 Table).

Saliva samples from 58 OPC cases and 20 controls were tested in the second set. Although 22 metabolites showed a difference (p < 0.05), none reached statistical significance when taking account of multiple comparisons (all FDR > 0.1) (S4 Table).

### Pathway analysis

**Fig 3:**
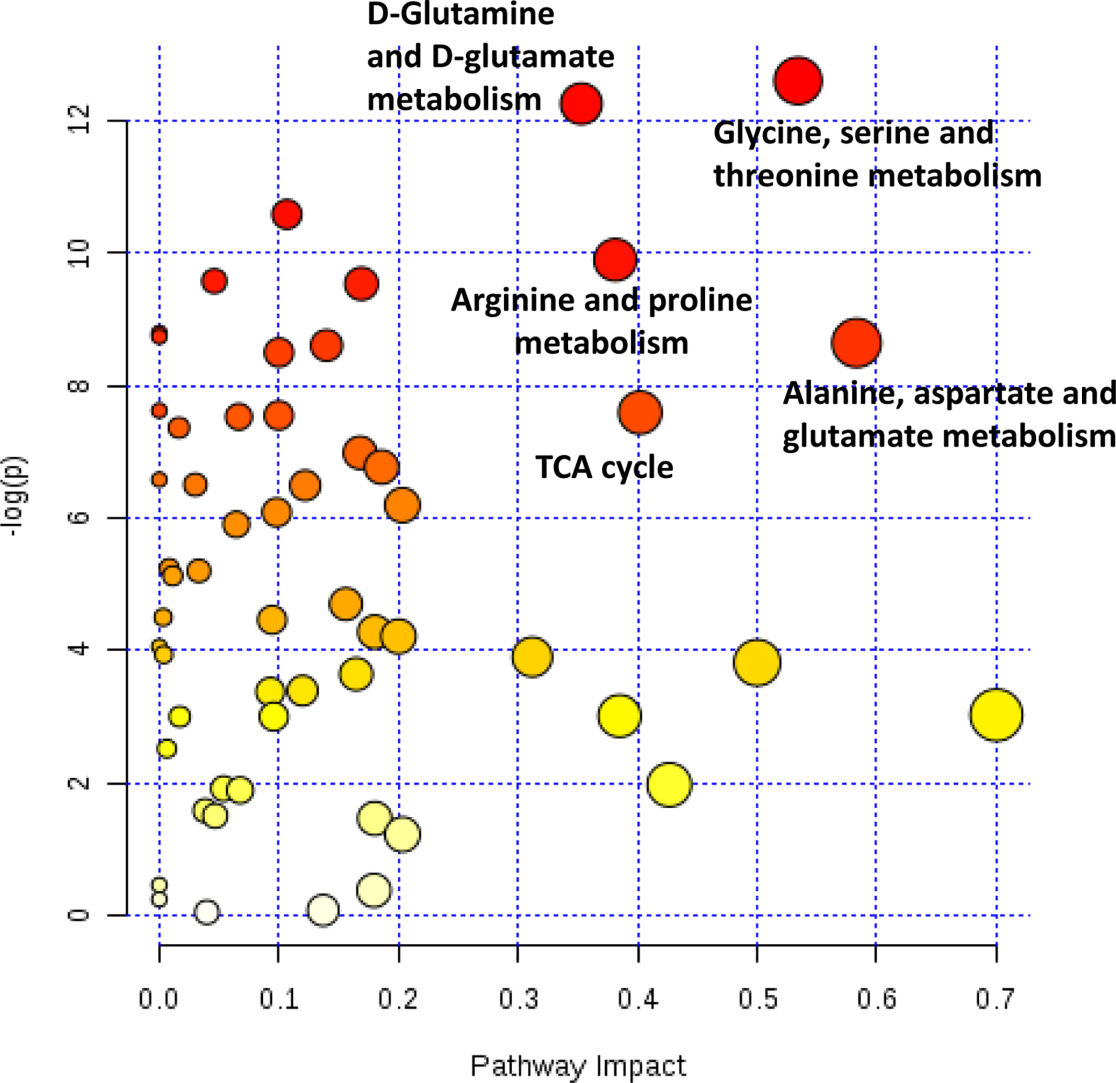
Pathway analysis results from MetaboAnalyst 3.0. The pathway analysis was based on 108 unique metabolites from Targeted LC-MS aqueous and NMR platforms. Y-axis is the -log p-values from pathway enrichment analysis. X-axis is the pathway impact values from pathway topology analysis. The node color and radius is based on its p-value and pathway impact values, respectively.

We used MetaboAnalyst 3.0 to examine the salivary metabolomics data. The 101 OCC patients and 35 controls from both the first and second sample sets combined were included in the pathway analysis to increase the sample size for analysis. Because only the NMR and targeted aqueous LC-MS data are comparable between the two sample sets, and many of the metabolites detected by the global platforms have no compound identification, we only used metabolites from NMR and the targeted platform for pathway analysis. Internal standard and duplicates metabolites between the two platforms were excluded, leaving 108 unique metabolites for pathway analysis. MetaboAnalyst takes into consideration both the number of detected metabolites in individual pathway and their alterations between cases and controls. We found five pathways with high significance and high pathway impact as judged by the MetaboAnalyst metrics. These included glycine, serine and threonine metabolism pathway; D-glutamine and D-glutamate metabolism pathway; arginine and proline metabolism pathway; alanine, aspartate and glutamate metabolism pathway; and the citric acid (TCA) cycle pathway (Fig 3). Pathway significance measures whether metabolites from a given pathway are over-represented in the 108 metabolites set compared to the total metabolites considered in the analysis. Pathway impact score measures whether the metabolites from the 108 metabolites set plays central roles in the metabolic network of a given pathway. Detailed results of the pathway analyses are presented in S5 Table.

## Discussion

In this salivary metabolomics study using independent sample sets of OCC cases and controls for discovery and validation, we found concentrations of glycine and proline in the saliva of OCC cases to be lower than that of controls in both sample sets. Moreover, both glycine and proline levels were consistently lower in OCC cases compared to controls assessed across different metabolomics assay platforms. Glycine plays a key role in one-carbon metabolism for the biosynthesis of purines, glutathione and histone methylation. While glycine is not an essential amino acid and can be synthesized from endogenous serine, results from a study by Jain et al. [48] of rapidly proliferating NCI-60 cancer cell lines showed that about a third of the intracellular glycine came from extracellular consumption and that consumed glycine was rapidly incorporated into purines. Furthermore, rapid cell proliferation was also accompanied by increased endogenous glycine synthesis via the mitochondrial glycine synthesis pathway. The mitochondrial glycine synthesis pathway involves the conversion of serine to glycine by serine hydroxymethyltransferase 2 (SHMT2), methylene tetrahydrofolate dehydrogenase (MTHFD2) and tetrahydrofolate synthetase (MTHFD1L), which generate cofactor tetrahydrofolate for the SHMT2 reaction. Other studies have also shown the important role of these mitochondrial enzymes in cancers [49–51]. Our previously generated transcriptomic data [35] (GSE30784) on tumor tissues of 115 OCC patients and 45 normal oral mucosa from controls (42 OCC and 19 controls from that study were also in the current study) showed that the transcript levels from OCC tissues for the mitochondrial *SHMT2* and *MTHFD2* for glycine synthesis were significantly higher in cases than in controls, suggesting there was increased glycine synthesis in the OCC tumor cells to meet the increasing demand for nucleotide synthesis in the rapidly proliferating cells. The respective p-values for case-control differences in the three Affymetrix probe IDs corresponding to the *SHMT2* gene (214095_at, 214096_s_at, and 214437_s_at) were <0.0001, 0.0387 and <0.0001. The p-values for case-control difference for *MTHFD2* were < 0.0001 for both 234976_x_at and 201761_at Affymetrix probe IDs. Thus, our results are consistent with the hypothesis that the OCC tumor cells are taking up glycine from the extracellular space bathed in saliva as well as actively synthesizing glycine in the mitochondria for the generation of one-carbon units for downstream nucleotide synthesis to support tumor progression. Further work would be needed to confirm or refute this hypothesis. For example, while the NCI-60 cancer cell line panel contains a large number of squamous lung cancer cell lines, it does not include cell lines of head and neck cancer. While lung cancer and head and neck cancer share tobacco-use as the primary etiologic factor, it nonetheless would be important to evaluate whether head and neck cancer cells actively consume extracellular glycine to increase the intracellular glycine pool for one-carbon metabolism and nucleotide synthesis.

Glycine, proline, ornithine, and citrulline were lower in stage I and II OCC compared to controls. Ornithine is a substrate for the synthesis of polyamines, citrulline, and proline by ornithine decarboxylase (ODC), ornithine transcarbomylase (OTC), and ornithine aminotransferase (OAT), respectively [52]. It is well established that an increase in polyamine synthesis by ODC is associated with several kinds of cancers, including head and neck cancer [53–55]. Our prior results showed that transcript levels for the *ODC1* gene (encoding the ODC enzyme for polyamine biosynthesis) was significantly upregulated in tumor tissues compared to controls whereas the transcript levels of *OTC* and *OAT* were downregulated in tumor tissue. The t-test p-values comparing tumors vs. controls for *ODC1* (Affymetrix probe ID 200790_at), *OTC* (Affymetrix probe ID 207200_at) and *OAT* (Affymetrix probe ID 201599_at) were < 0.0001, 0.0285, and <0.0001 respectively. When taken together, these results suggest that, in OCC, ornithine might be used for synthesis of polyamines more than for the synthesis of citrulline or proline, and may partly explain why we found lower levels of salivary citrulline and proline in OCC patients.

Based on the pathway analysis, we found five cellular pathways to be highly associated with OCC. Two of these pathways (the alanine, aspartate and glutamate metabolism pathway and the arginine and proline metabolism pathway) were reported to be associated with mutant p53 status [56], which is in line with our knowledge that p53 mutation is a frequent event in OCC [57–59]. Dysregulation of glutamine metabolism and TCA cycle have also been associated with several types of cancers [60–64].

A limitation of our study is the relatively small sample size of the validation set which may limit the power to distinguish OCC from controls. Although only two of 80 metabolites were significant in both the discovery and validation sets, we found some of the 80 metabolites overlapping with salivary metabolites differentiating oral cancer and controls reported by other studies. For example, using ultraperformance liquid chromatography (UPLC) coupled with Q TOF-MS, Wei et al. [34] reported a panel of five metabolites (γ-aminobutyric acid, phenylalanine, valine, n-neicosanoic acid and lactic acid) capable of discriminating oral cancer (n = 37) from controls (n = 34) or OLK (n = 32) among Chinese in Shanghai. Three of the five metabolites (phenylalanine, valine, and lactic acid) were among our 80 metabolites differentiating between OCC and controls. Moreover, in our study, the concentrations of lactic acid and valine were found to be significantly different between cases and controls as measured by more than one platform. A small study from western China of 30 OSCC and 30 controls by Wang et al. [65] reported 14 salivary metabolites as potential biomarkers for diagnosis of OSCC. Among these 14 metabolites, three (ornithine, succinic acid, and lactic acid) were among our 80 metabolites. However, they found salivary ornithine to be higher in cancer patients, while we found lower levels of salivary ornithine in OCC patients compared to controls.

There have been fewer than a dozen publications on salivary metabolomics employing a variety of platforms comparing oral cancer and controls. Our findings are somewhat inconsistent with some published reports. Using capillary electrophoresis mass spectrometry (CE-MS), Sugimoto et al. [33] found 28 salivary metabolites that discriminate oral cancer cases (n = 69) from controls (n = 87). Although several of our 80 metabolites overlapped with their 28 metabolites, the direction of up- or down-regulation of metabolites in saliva samples of cancer patients compared to controls is not consistent between our study and their study. In addition, their results showed no significant difference between cases and controls for glycine and proline (p-values 1.0 and 0.968, respectively). Ohshima et al. [66] compared 22 Japanese OSCC cases and 21 controls using CE-MS reported 25 salivary metabolites to be significantly different in their levels. However, glycine and proline were not among them. No comparison of our results with those of an earlier study by Yan et al. [30] can be made, since their results, based on HPLC-MS retention times, did not provide identities of the peaks showing differences when comparing saliva from 20 OSCC, 7 OLK and 11 controls. These inconsistent results can be due to a number of factors, such as the difference in metabolomics platforms that may have different sensitivity and specificity, the difference in study population, tumor site, saliva collection/preservation methods, sample size, and statistical methods. Most of these studies are quite small and did not employ a training and testing sets to confirm their own discoveries. Another factor that may contribute to the inconsistency in the results is the comparability of the microbial community among the study participants in the various studies. It is highly likely that the types and levels of salivary metabolites are strongly influenced by the types of oral microbes present in the oral cavity. Oral microbiome differences can be attributed to genetics, race-ethnicity, geography, dietary patterns, etc. [67–72]. The majority of the published studies were based on participants of Asian origin, only Sugimoto et al. was based on study participants in the US. Moreover, the duration of fasting could potentially affect the result and a longer fasting time may facilitate the discovery of a greater number of markers with discriminatory potential, as described by Ishikawa et al [73].

Of the four analytical platforms used in this study, the targeted LC-MS/MS and NMR methods were quite stable. Our first and second set of samples were tested almost one year apart but we still see a good correlation between results of the same samples that were tested in both sets. Demonstrated reproducibility is of critical importance for a biomarker test. In our experience, it is quite a challenge to match the two datasets generated from global profiling, partly due to a large number of unknown features in the global metabolomics data sets and the sensitivity of the global platforms to small changes in instrument performance, column conditions and other experimental factors [74]. Not having gold standards to calibrate the various approaches and instruments used in metabolomics research is clearly a hindrance in making comparisons of studies, and an important area for the field of metabolomics to improve upon.

To our knowledge, there is no publication on salivary metabolites comparing OCC patients with and without nodal metastasis. Thus, we could not compare our results to others. It is possible that there is truly no difference between salivary metabolite levels measured in this study between patients with different nodal status or the difference is so subtle that a very large sample size will be needed for investigation.

## Conclusions

We found salivary glycine and proline to be lower in oral cavity cancer patients compared to controls but did not find a difference in the concentration of salivary metabolites between oral cavity cancer patients with and without nodal metastasis, or between oropharyngeal cancer patients and controls. A larger sample size may be needed to confirm these results.

## Supporting information

S1 Fig Typical 800 MHz 1H NMR spectrum of a saliva sample with labeling of most of the metabolite peaks, which were used for obtaining their relative concentrations. (TIF)

S1 Table Raw data of the four metabolomics tests for the first set of samples. (XLSX)

S2 Table Raw data of the four metabolomics tests for the second set of samples. (XLSX)

S3 Table Data of both sample sets after filtering and normalization. (XLSX)

S4 Table Results of linear regression analyses comparing salivary metabolites from oral cavity cancer (OCC) patients with controls, OCC with and without nodal metastasis, and oropharyngeal cancer vs. controls. (XLSX)

S5 Table Pathways associated with oral cavity cancer status from MetaboAnalyst 3.0. (XLSX)
