# Supplementary figures and images for: Salivary metabolite profiling distinguishes patients with oral cavity squamous cell carcinoma from normal controls
Source: PLoS One. 2018 Sep 20;13(9):e0204249. doi: 10.1371/journal.pone.0204249 (PMC6147497; doi:10.1371/journal.pone.0204249)

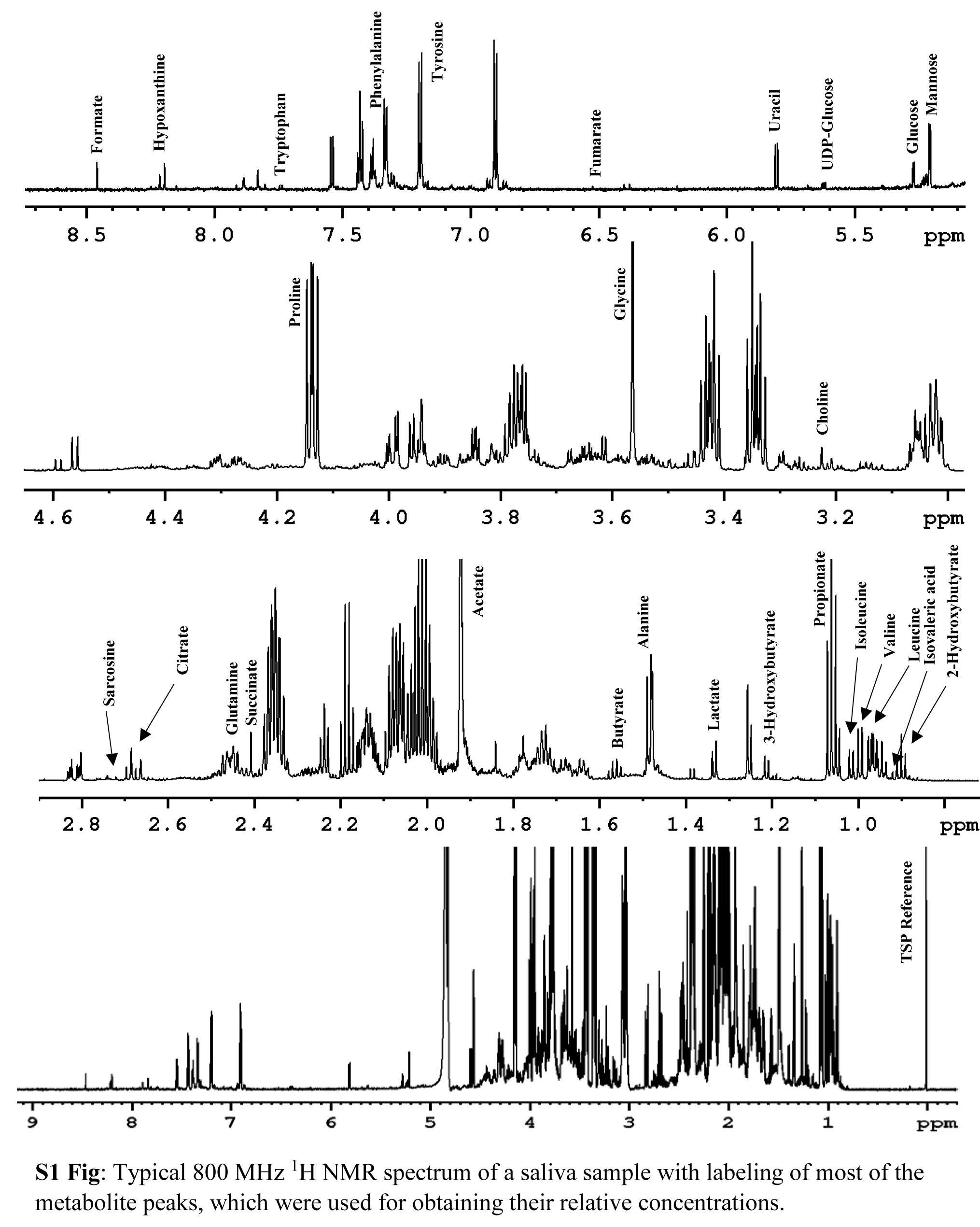

Supplement: S1 Fig — (TIF) [file pone.0204249.s001.tif]
